# Objectively-measured movement behaviors, systemic low-grade inflammation, and plasma neurofilament light chain in older adults: a population-based study

**DOI:** 10.1186/s12979-023-00363-7

**Published:** 2023-07-25

**Authors:** Yongxiang Wang, Qi Han, Xiaolei Han, Yi Dong, Ming Mao, Chaoqun Wang, Xiaojie Wang, Shi Tang, Cuicui Liu, Yuanjing Li, Tingting Hou, Lin Cong, Yifeng Du, Chengxuan Qiu

**Affiliations:** 1 Department of Neurology, Shandong Provincial Hospital affiliated to Shandong First Medical University, No. 324, Jingwu Road, Jinan, Shandong People’s Republic of China; 2 Institute of Brain Science and Brain-Inspired Research, Shandong First Medical University & Shandong Academy of Medical Sciences, Jinan, Shandong People’s Republic of China; 3 Department of Neurology, Shandong Provincial Hospital, Shandong University, Jinan, Shandong People’s Republic of China; 4 Key Laboratory of Endocrine Glucose & Lipids Metabolism and Brain Aging in Shandong First Medical University, Ministry of Education of the People’s Republic of China, Jinan, Shandong People’s Republic of China; 5 Aging Research Center and Center for Alzheimer Research, Department of Neurobiology, Care Sciences and Society, Karolinska Institutet-Stockholm University, Karolinska Institutet, Tomtebodavägen 18A, 171 65 Solna, Stockholm, Sweden

**Keywords:** Accelerometer, Movement behaviors, Neurofilament light chain, Low-grade inflammation, Population-based study

## Abstract

**Background:**

Evidence has linked self-reported sedentary behavior (SB) and physical activity (PA) with cognitive impairment; however, the underlying mechanisms are poorly understood. We examined the associations of the accelerometer-measured movement behaviors with plasma neurofilament light chain (NfL) among older adults and the role of systemic low-grade inflammation in the associations.

**Results:**

This population-based study included 1,029 dementia-free older adults (age ≥ 60 years, range 60–88 years; 59.48% women) who undertook the ActiGraph substudy (March 2018-December 2020) in MIND-China. There were nonlinear relationships of daily SB and PA time with plasma NfL concentration, such that more daily SB time or less time spent in daily light-intensity physical activity (LPA) and moderate-to-vigorous-intensity physical activity (MVPA) was significantly associated with increased plasma NfL only when SB time ≥ 8.00 h/day or LPA time < 5.00 h/day or MVPA time < 2.00 h/day. Furthermore, more daily SB time or less daily LPA and MVPA time was significantly associated with higher serum low-grade inflammation score, a composite measure generated from serum IL-6, IL-8, TNF-α, and ICAM-1 (*P* < 0.05). Finally, low-grade inflammation score accounted for 14.5% to 17.8% of the associations between movement behaviors and plasma NfL.

**Conclusions:**

More daily SB and less PA time are associated with neurodegeneration and systemic low-grade inflammation in older adults. The association of movement behaviors with neurodegeneration is partially mediated by low-grade inflammation.

## Background

Epidemiological studies have consistently shown that sedentary behavior (SB) is associated with increased risks of cardiovascular morbidity and mortality [1, 2] and that moderate intensity physical activity (PA) could partly eliminate the risk of mortality due to SB [3]. In addition, evidence has accumulated that movement behaviors (e.g., SB and PA) are associated with cognitive function in older adults. Specifically, SB has been associated with an increased risk of cognitive impairment and dementia, while PA may facilitate neuroplasticity of certain brain structures and aspects of cognition [4, 5]. However, the pathophysiological mechanisms underlying the associations of movement behaviors with cognition remain poorly understood. Sedentary lifestyle could be linked with cognitive impairment via damages to cardiovascular and metabolic systems such as cerebral macro- and microvascular diseases [6], cerebral hypoperfusion [7], and structural brain lesions [8]. Alternatively, SB may affect cognitive function via influencing neurodegenerative lesions in the brain, a pathway that has been rarely investigated in studies of the general population settings, partly due to infeasibility of measuring biomarkers in the central nervous system (CNS) (e.g., cerebrospinal fluid [CSF] and brain Positron Emission Tomography [PET]).

Population-based cross-sectional studies have suggested that self-reported habitual PA is related to the reduced amyloid load in the brain and increased CSF amyloid beta 42 (Aβ42) [9, 10]. In addition, higher levels of self-reported PA were associated with lower plasma Aβ40 and Aβ42 levels in cognitively normal older adults [11]. Notably, most of the previous studies have mainly investigated the relationship of movement behaviors with CNS or peripheral biomarkers for Alzheimer’s disease (AD) (e.g., Aβ), and no population-based studies have examined the associations with peripheral biomarkers for neurodegeneration. In recent years, plasma neurofilament light chain (NfL) protein has emerged as a promising biomarker for neurodegeneration in several neurological diseases (e.g., AD and multiple sclerosis) [12]. This provides a potential to investigate the relationships of movement parameters with neurodegeneration in the large-scale general population settings.

Furthermore, most of the previous studies have assessed SB and PA parameters using the self-reported questionnaires, which are prone to recall bias and misclassification of movement behaviors, and could potentially lead to biased associations [13]. ActiGraph accelerometry could objectively quantify various movement parameters, such as daily time spent in SB, light-intensity physical activity (LPA), and moderate-to-vigorous-intensity physical activity (MVPA) [14]. This is crucial to explore the relationships of SB and PA parameters with peripheral biomarkers of neurodegeneration.

In addition, robust evidence supports that both brain neuroinflammation and peripheral low-grade inflammation (e.g., interleukin-6 [IL-6]; interleukin-8 [IL-8], and tumor necrosis factor alpha [TNF-α]) are involved in the pathogenesis and progression of neurodegeneration and AD [15–17]. Data from a subsample of the Ginkgo Evaluation of Memory Study of cognitively unimpaired older adults showed that peripheral inflammatory biomarkers (e.g., IL-6 and C-reactive protein) were correlated with brain biomarkers of Aβ deposition [18]. Moreover, observational studies consistently report an association between regular PA and lower systemic inflammation [19]. By contrast, more sedentary time and prolonged sedentary bouts were associated with high levels of low-grade inflammation [20]. However, the association of objectively-measured daily PA and SB with peripheral biomarkers for neurodegeneration and systemic low-grade inflammation has not been thoroughly explored in the general population settings.

Therefore, in this population-based study, we aimed to systematically examine the interrelationships of accelerometer-measured daily SB and PA times with plasma NfL and low-grade inflammatory biomarkers (e.g., IL-6, IL-8, TNF-a, and intercellular cell adhesion molecule-1 [ICAM-1]) among rural-dwelling dementia-free older adults in China. Our hypothesis was that movement behaviors (e.g., SB and PA) were associated with peripheral neurodegenerative biomarker in older adults and that low-grade inflammation may play a part in the associations.

## Results

### Characteristics of the study participants

The average age of the 1,029 participants was 69.56 (SD, 4.47; age range, 60–88) years, 59.48% were females, and 36.73% were illiterate (Table 1). Compared with men, women were less likely to be educated, smoke, and consume alcohol, and more likely to be obese and have a history of coronary heart disease, diabetes, and dyslipidemia (all *P* < 0.05). Overall, participants spent an average of 8.16 h of daily total ActiGraph wear time on SB (57.59%), 4.93 h on LPA (34.80%), and 1.08 h on MVPA (7.62%). Women spent less time than men in SB, but more time in LPA and MVPA (all *P* < 0.001) (Table 1). In addition, compared to men, women had higher serum ICAM-1 concentration (*P* = 0.002), but lower plasma NfL concentration (*P* < 0.001). There were no significant sex differences in mean age, the distribution of *APOE* ε4 allele, stroke, hypertension, daily ActiGraph wear time, and serum concentrations of IL-6, IL-8, and TNF-α (Table 1).

**Table 1:** Characteristics of the study participants by sex (*n* = 1,029)

Characteristics	Total sample (*n* = 1,029)	Men (*n* = 417)	Women (*n* = 612)	*P*-value
Age range (years)	60–88	60–84	60–88	–
Age, years	69.56 (4.47)	69.69 (4.47)	69.47 (4.48)	0.472
Education, n (%)				** < 0.001**
Illiterate	378 (36.73)	40 (9.59)	338 (55.23)	
Primary school	446 (43.34)	209 (50.12)	237 (38.73)	
Middle school or above	205 (19.92)	168 (40.29)	37 (6.05)	
Alcohol intake, n (%)				** < 0.001**
Never	632 (61.42)	71 (17.03)	561 (91.67)	
Former	82 (7.97)	75 (17.99)	7 (1.14)	
Current	315 (30.61)	271 (64.99)	44 (7.19)	
Smoking, n (%)				** < 0.001**
Never	681 (66.18)	82 (19.66)	599 (97.88)	
Former	147 (14.29)	140 (33.57)	7 (1.14)	
Current	201 (19.53)	195 (46.76)	6 (0.98)	
Obesity, n (%)	214 (20.80)	72 (17.27)	142 (23.20)	**0.021**
*APOE* ε4 allele, n (%)^a^	160 (15.55)	64 (15.35)	96 (15.69)	0.758
Stroke, n (%)	133 (12.93)	59 (14.15)	74 (12.09)	0.334
Coronary heart disease, n (%)	211 (20.51)	60 (14.39)	151 (24.67)	** < 0.001**
Hypertension, n (%)	731 (71.04)	299 (71.70)	432 (70.59)	0.699
Diabetes, n (%)	158 (15.35)	49 (11.75)	109 (17.81)	**0.008**
Dyslipidemia, n (%)	247 (24.00)	66 (15.83)	181 (29.58)	** < 0.001**
Wear seasons, n (%)				**0.036**
Spring	167 (16.23)	68 (16.31)	99 (16.18)	
Summer	385 (37.41)	153 (36.69)	232 (37.91)	
Autumn	398 (38.68)	152 (36.45)	246 (40.20)	
Winter	79 (7.68)	44 (10.55)	35 (5.72)	
Daily ActiGraph wear time, hours	14.17 (1.35)	14.08 (1.38)	14.23 (1.33)	0.117
Daily SB time, hours	8.16 (1.92)	8.84 (1.87)	7.71 (1.81)	** < 0.001**
Daily LPA time, hours	4.93 (1.48)	4.37 (1.47)	5.32 (1.36)	** < 0.001**
Daily MVPA time, hours	1.08 (0.84)	0.88 (0.69)	1.21 (0.90)	** < 0.001**
NfL, pg/ml, median (IQR)	11.98 (6.60)	12.99 (7.29)	11.26 (6.20)	** < 0.001**
IL-6, pg/ml, median (IQR)	0.86 (0.68)	0.86 (0.67)	0.85 (0.67)	0.584
IL-8, pg/ml, median (IQR)	17.30 (20.35)	16.21 (17.40)	18.39 (22.28)	**0.027**
TNF-α, pg/ml, median (IQR)	1.73 (0.59)	1.71 (0.58)	1.74 (0.60)	0.457
ICAM-1, ng/ml, median (IQR)	478.83 (100.52)	466.93 (100.65)	486.92 (99.71)	**0.002**

Data were mean (SD), unless otherwise specified

*Abbreviations*: *APOE* Apolipoprotein E gene, *SB* Sedentary behavior, *LPA* Light-intensity physical activity, *MVPA* Moderate-to-vigorous-intensity physical activity, *NfL* Neurofilament light chain, *IQR* Interquartile range, *IL-6* Interleukin-6, *IL-8* Interleukin-8, *TNF-α* Tumor necrosis factor alpha, *ICAM-1* Intercellular cell adhesion molecule-1

^a^There were 13 participants who had missing information on *APOE* genotype and a dummy variable was created to represent these individuals in the subsequent analyses

### Associations of movement behaviors with plasma NfL (*n* = 1029)

**Fig. 1:**
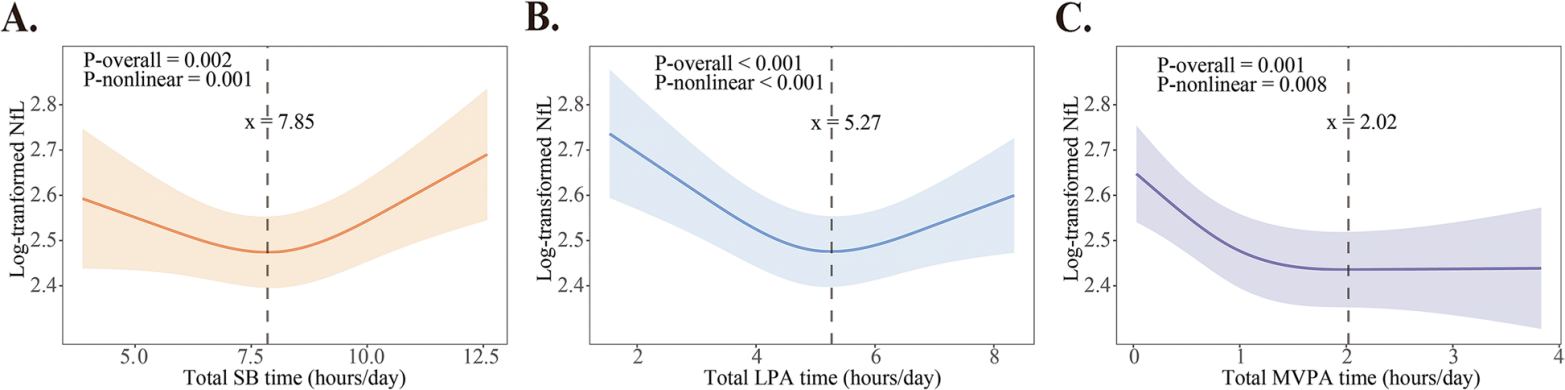
Continuous dose-response associations of daily SB (**A**), LPA (**B**), and MVPA (**C**) time with plasma NfL concentration fitted by restricted cubic spline models. The raw data of plasma NfL were natural log-transformed due to skewed distributions. The solid lines and gray areas represent the estimated values and 95% confidence intervals of biomarkers, respectively. The analyses were adjusted for age, sex, education, ActiGraph wear seasons and daily wear time, smoking, alcohol intake, APOE ε4 allele, obesity, hypertension, dyslipidemia, diabetes, stroke, and coronary heart disease. In addition, the analyses of MVPA and LPA were mutually adjusted for each other and the analyses of SB were adjusted for MVPA. Abbreviations: SB, sedentary behavior; LPA, light-intensity physical activity; MVPA, moderate-to-vigorous-intensity physical activity; NfL, neurofilament light chain

The restricted cubic spline (RCS) analysis suggested nonlinear relationships of daily SB, LPA, and MVPA times with plasma NfL (all *P*-overall < 0.05, *P*-nonlinear < 0.05, Fig. 1). Thus, we used the dose–response trajectory analysis to further investigate the nonlinear relationships of movement behaviors with plasma NfL level. The inflection points (rounded to an integer) for the associations of daily SB, LPA, and MVPA time with plasma NfL were 8.00, 5.00, and 2.00 h, respectively. Specifically, there was no significant association between daily SB time and plasma NfL on the left of inflection point; however, on the right of inflection point (daily SB time ≥ 8.00 h), the multivariable-adjusted β-coefficient (95% confidential interval [CI]) of plasma NfL concentration (natural log-transformed) associated with per 1-h increase in daily SB time was 0.049 (0.005, 0.093). By contrast, more time spent in daily LPA and MVPA was significantly associated with lower plasma NfL when daily LPA time was < 5.00 h and daily MVPA time was < 2.00 h, whereas there was no significant association of either daily LPA time or daily MVPA time with plasma NfL on the right of inflection point (Table 2).

**Table 2:** Associations of accelerometer-assessed movement behaviors with plasma NfL concentration by daily sedentary behavior or physical activity time (*n* = 1,029)

Behavioral factors	β-coefficient (95% confidence interval), plasma NfL (pg/ml), log-transformed		
	**Model 1**^a^	**Model 2**^a^	**Model 3**^a^
**Daily SB time, hours**			
< 8.00 (*n* = 478)	-0.008 (-0.043, 0.027)	-0.005 (-0.040, 0.030)	-0.041 (-0.086, 0.004)
≥ 8.00 (*n* = 551)	**0.072 (0.037, 0.108)**^**‡**^	**0.060 (0.025, 0.095)**^†^	0.049 (0.005, 0.093)^*^
**Daily LPA time, hours**			
< 5.00 (*n* = 530)	**-0.112 (-0.158, -0.066)**^**‡**^	**-0.091 (-0.137, -0.044)**^**‡**^	**-0.073 (-0.123, -0.023)**^**†**^
≥ 5.00 (*n* = 499)	0.027 (-0.020, 0.073)	0.033 (-0.013, 0.079)	0.038 (-0.008, 0.084)
**Daily MVPA time, hours**			
< 2.00 (*n* = 902)	**-0.123 (-0.183, -0.063)**^**‡**^	**-0.119 (-0.179, -0.060)**^**‡**^	**-0.108 (-0.175, -0.041)**^**†**^
≥ 2.00 (*n* = 127)	-0.067 (-0.184, 0.050)	-0.079 (-0.201, 0.042)	-0.087 (-0.210, 0.036)

^a^Model 1 was adjusted for age, sex, education, wear seasons, and daily wear time of ActiGraph; Model 2 was additionally adjusted for smoking, alcohol intake, *APOE* ε4 allele, obesity, hypertension, dyslipidemia, diabetes, stroke, and coronary heart disease; and in Model 3, the analyses of MVPA and LPA were mutually adjusted for each other and the analyses of SB were adjusted for MVPA

*Abbreviations*: *SB* Sedentary behavior, *LPA* Light-intensity physical activity, *MVPA* Moderate-to-vigorous-intensity physical activity, *NfL* Neurofilament light chain

^*^P < 0.05, ^†^*P* < 0.01, ^‡^*P* < 0.001

### Associations of movement behaviors with serum biomarkers of low-grade inflammation (*n* = 991)

Controlling for demographics and wear seasons and daily wear time of ActiGraph, the β-coefficients (95% CI) of the composite score of low-grade inflammatory biomarkers associated with daily SB, LPA, and MVPA time were 0.05 (0.02, 0.07), -0.06 (-0.09, -0.03), and -0.08 (-0.13, -0.03), respectively, and the associations remained statistically significant in model 2 when additionally adjusting for lifestyles, cardiometabolic risk factors, and *APOE* genotype (Table 3). However, the associations became statistically non-significant in model 3 when movement parameters were mutually controlled for each other. The association patterns of individual z-scores of IL-6, IL-8, and TNF-α with ActiGraph parameters were similar to those with the composite score of low-grade inflammation. By contrast, daily time spent in SB, LPA, and MVPA was not significantly related to serum ICAM-1 concentration (Table 3).

**Table 3:** Associations of accelerometer-assessed movement behaviors with serum biomarkers of systemic low-grade inflammation (*n* = 991)

Behavioral factors	β-coefficient (95% confidence interval), z scores of serum cytokines		
	**Model 1**^**#**^	**Model 2**^**#**^	**Model 3**^**#**^
**IL-6, z-score**			
Daily SB time, hours	**0.06 (0.03, 0.10)**^**‡**^	**0.06 (0.02, 0.09)**^**†**^	0.04 (-0.01, 0.10)
Daily LPA time, hours	**-0.07 (-0.12, -0.02)**^**†**^	-0.06 (-0.11, -0.01)^*^	-0.04 (-0.10, 0.01)
Daily MVPA time, hours	**-0.13 (-0.21, -0.05)**^**†**^	-0.10 (-0.18, -0.02)^*^	-0.08 (-0.16, 0.01)
**IL-8, z-score**			
Daily SB time, hours	0.05 (0.01, 0.09)^*^	0.05 (0.01, 0.09)^*^	0.05 (-0.01, 0.11)
Daily LPA time, hours	-0.06 (-0.12, -0.01)^*^	-0.06 (-0.12, -0.01)^*^	-0.05 (-0.11, 0.01)
Daily MVPA time, hours	-0.07 (-0.15, 0.02)	-0.08 (-0.16, -0.01)	-0.05 (-0.14, 0.04)
**TNF-α, z-score**			
Daily SB time, hours	**0.06 (0.02, 0.09)**^**†**^	0.05 (0.01, 0.08)^*^	0.03 (-0.02, 0.09)
Daily LPA time, hours	**-0.07 (-0.11, -0.02)**^**†**^	-0.05 (-0.10, 0.00)^*^	-0.03 (-0.09, 0.02)
Daily MVPA time, hours	-0.10 (-0.18, -0.02)^*^	-0.10 (-0.17, -0.02)^*^	-0.08 (-0.16, 0.01)
**ICAM-1, z-score**			
Daily SB time, hours	0.02 (-0.02, 0.06)	0.01 (-0.02, 0.05)	0.04 (-0.02, 0.10)
Daily LPA time, hours	-0.04 (-0.09, 0.01)	-0.03 (-0.08, 0.02)	-0.04 (-0.10, 0.02)
Daily MVPA time, hours	-0.01 (-0.09, 0.07)	0.01 (-0.07, 0.09)	0.04 (-0.05, 0.12)
**Low-grade inflammation, composite score**			
Daily SB time, hours	**0.05 (0.02, 0.07)**^**‡**^	**0.04 (0.02, 0.07)**^**†**^	0.04 (0.01, 0.08)^*^
Daily LPA time, hours	**-0.06 (-0.09, -0.03)**^**‡**^	**-0.05 (-0.09, -0.02)**^**†**^	-0.04 (-0.08, -0.01)^*^
Daily MVPA time, hours	**-0.08 (-0.13, -0.03)**^**†**^	-0.07 (-0.12, -0.01)^*^	-0.04 (-0.10, 0.02)

^#^Model 1 was adjusted for age, sex, education, wear seasons, and daily wear time of ActiGraph; Model 2 was additionally adjusted for smoking, alcohol intake, *APOE* ε4 allele, obesity, hypertension, dyslipidemia, diabetes, stroke, and coronary heart disease; and in Model 3, the analyses of MVPA and LPA were mutually adjusted for each other and the analyses of SB were adjusted for MVPA

*Abbreviations*: *SB* Sedentary behavior, *LPA* Light physical activity, *MVPA* Moderate-to-vigorous intensity physical activity, *IL-6* Interleukin-6, *IL-8* Interleukin-8, *TNF-α* Tumor necrosis factor alpha, *ICAM-1* Intercellular cell adhesion molecule-1

^*^P < 0.05, ^†^*P* < 0.01, ^‡^*P* < 0.001

### Mediation of low-grade inflammation in the associations of movement parameters with plasma NfL (*n* = 991)

In the mediation modeling analysis, the composite score of low-grade inflammatory biomarkers significantly mediated the associations of daily SB, LPA, and MVPA with plasma NfL, with the mediating effects being 0.005 (95% CI: 0.002, 0.010), -0.007 (-0.013, -0.003), and -0.009 (-0.018, -0.003), respectively (Table 4). The corresponding proportions of mediation were 16.1%, 17.8%, and 14.5%, respectively.

**Table 4:** Mediation effect of low-grade inflammation on the association of movement behaviors with plasma NfL concentration (*n* = 991)

Parameters from mediation models	Estimated β-coefficient (95% confidence interval)
**Association of SB with plasma NfL**	
Total effect	**0.033 (0.015, 0.052)**^‡^
Mediation effect by inflammation score	**0.005 (0.002, 0.010)**^‡^
Direct effect	**0.028 (0.010, 0.047)**^†^
Proportion mediated	**0.161 (0.064, 0.396)**^‡^
**Association of LPA with plasma NfL**	
Total effect	**-0.038 (-0.065, -0.014)**^†^
Mediation effect by inflammation score	**-0.007 (-0.013, -0.003)**^‡^
Direct effect	-0.031 (-0.057, -0.008)^*^
Proportion mediated	**0.178 (0.061, 0.484)**^†^
**Association of MVPA with plasma NfL**	
Total effect	**-0.061 (-0.097, -0.027)**^‡^
Mediation effect by inflammation score	**-0.009 (-0.018, -0.003)**^†^
Direct effect	**-0.052 (-0.087, -0.016)**^†^
Proportion mediated	**0.145 (0.041, 0.371)**^†^

Mediation analysis was adjusted for age, sex, education, and wear seasons and daily wear time of ActiGraph

*Abbreviations*: *SB* Sedentary behavior, *LPA* Light physical activity, *MVPA* Moderate-to-vigorous intensity physical activity, *NfL* Neurofilament light chain

^*^P < 0.05, ^†^*P* < 0.01, ^‡^*P* < 0.001

## Discussion

In this large-scale population-based cross-sectional study, we systematically investigated the dose–response associations of accelerometer-measured movement behaviors with peripheral biomarkers for neurodegeneration and systemic low-grade inflammation among dementia-free older adults living in rural communities. The main findings can be summarized as follows: (1) there were nonlinear relationships of daily SB and PA time with plasma NfL concentration, depending on daily time spent in PA and SB. Specifically, more SB time (inflection point, 8.00 h/day), less LPA time (inflection point, 5.00 h/day), and less MVPA time (inflection point, 2.00 h/day) were associated with increased plasma NfL; (2) more daily SB time was associated with a higher score of low-grade inflammation, while PA of different intensities was associated with a reduced score of low-grade inflammation; and (3) the associations of movement behaviors with plasma NfL were partially mediated by low-grade inflammation.

Population-based studies have rarely investigated the relationship between objectively-measured SB and PA time and plasma neurodegenerative biomarkers among older adults. Our community-based study of dementia-free older adults revealed nonlinear relationships of daily time spent in SB, LPA, and MVPA with plasma NfL. Specifically, plasma NfL remained relatively stable with increase in daily SB time up to 8 h, and then linearly increased with increasing daily SB time, whereas plasma NfL decreased with increasing daily time spent in LPA and MVPA up to 5.00 h and 2.00 h, respectively, thereafter, plasma NfL reached a plateau with increases in daily PA time. This relationship pattern between daily SB and PA time and plasma NfL have not yet been reported previously, but are in line with the report from a large-scale consortium study of late-life PA time in association with the risk of incident dementia [21]. Indeed, as people age, daily time spent in SB gradually increases and time spent in PA and intensity of PA decrease due to the age-related reduction in functional capacity of the cardiorespiratory and muscular systems. Furthermore, the greater the daily time and intensity of PA, the greater the risk of potential injury among older people. Therefore, there might be thresholds for daily time and intensity of PA for older adults that above which spending more time in PA may not add further benefits. However, the relationship patterns between movement behaviors and plasma NfL deserve further investigation in different populations.

The findings from our accelerometer-based studies were largely consistent with some reports on the associations of self-reported movement behaviors with neurodegenerative biomarkers. For example, the French Multidomain Alzheimer’s Preventive Trial observed a cross-sectional association of self-reported high PA with low plasma NfL [22]. The cross-sectional data from the Australian Imaging, Biomarkers and Lifestyle (AIBL) study showed an association between self-reported PA and PET-quantified brain tau burden [23]. However, several small-scale studies that examine the relationships of SB and PA with brain amyloid load have yielded mixed results. For instance, the Harvard Aging Brain Study observed that the pedometer-measured PA time was not associated with brain Aβ burden among clinically normal older adults [24]. By contrast, the Wisconsin Registry for Alzheimer's Prevention of cognitively healthy late middle-aged adults suggested that sedentariness was associated with a greater CSF Aβ burden, whereas moderate PA, but not light or vigorous PA, was associated with a favorable profile of CSF biomarkers for AD (e.g., higher Aβ42, lower total tau/Aβ42 ratio, and lower phosphorylated-tau/Aβ42 ratio) [25, 26]. In addition, data from AIBL showed an association of higher PA levels with lower plasma Aβ40 and Aβ42 levels only among *APOE* ε4 non-carriers [11]. The discrepant findings might be partially due to differences in assessment methodology (e.g., movement behaviors were assessed via self-reported questionnaire vs. objective pedometer) and characteristics of the study population (e.g., age, socioeconomic status, and genetic susceptibility).

The mechanisms linking movement behaviors to plasma neurodegenerative biomarkers are not fully understood. Previous studies suggested that sedentary lifestyle might lead to accumulation of visceral fat and reduction of blood flow, thus increasing release of cytokines and inducing a low-grade inflammatory state, which is associated with the development of atherosclerosis [27], insulin resistance [28], and neurodegeneration [29]. Conversely, PA is thought to ameliorate systemic low-grade inflammation [19]. In line with this hypothesis, our study did show that higher daily SB time was associated with increased serum IL-6, IL-8, TNF-α, and systemic low-grade inflammation, whereas higher daily LPA and MVPA time was related to reduced serum levels of multiple cytokines and systemic low-grade inflammation. Notably, our study revealed for the first time that serum inflammatory biomarkers could partially mediate the associations of movement behaviors with plasma neurodegenerative biomarker (NfL), with the proportion of mediation being around 15%. This suggests that sedentary lifestyle might contribute to neurodegeneration partly via inducing systemic low-grade inflammation.

A marked strength of this study was the large-scale population-based study that engaged older adults living in rural communities in China, a sociodemographic group that has been greatly underrepresented in research of movement behaviors and health. In addition, we employed the validated objective methods to assess movement parameters and the ultrasensitive single molecule array (SIMOA) technology to detect plasma biomarkers. Our study also has limitations. Accelerometers cannot distinguish completely activities between sitting and standing postures, which could potentially classify the time of both postures as sedentary time. In addition, there was a time interval between collection of plasma (March-September 2018) and ActiGraph assessment (August 2018-December 2020), which should be kept in mind when interpreting the results. Finally, our cross-sectional design does not allow us to infer a causal relationship for any of the observed associations of movement parameters with plasma neurodegenerative and inflammatory biomarkers.

## Conclusion

In summary, this population-based study of rural-dwelling older adults provides strong evidence that objectively-measured more SB time and less PA time were associated with unfavorable profile of peripheral biomarkers for neurodegeneration and systemic low-grade inflammation. We further revealed that the association between movement behaviors (SB and PA) and plasma NfL was partially mediated by low-grade inflammation. These findings have significant implications for understanding the potential pathophysiological mechanisms linking SB and PA with cognitive phenotypes in older adults. Future longitudinal studies are warranted to further clarify the potential causal relationships of SB and PA with biomarkers of brain aging and neurodegeneration and the potential role of systemic low-grade inflammation in their relationships. This will facilitate the development of preventive interventions to promote healthy brain aging and delay the onset of the neurodegenerative disorders (e.g., AD) among elderly people.

## Material and methods

### Study population

**Fig. 2:**
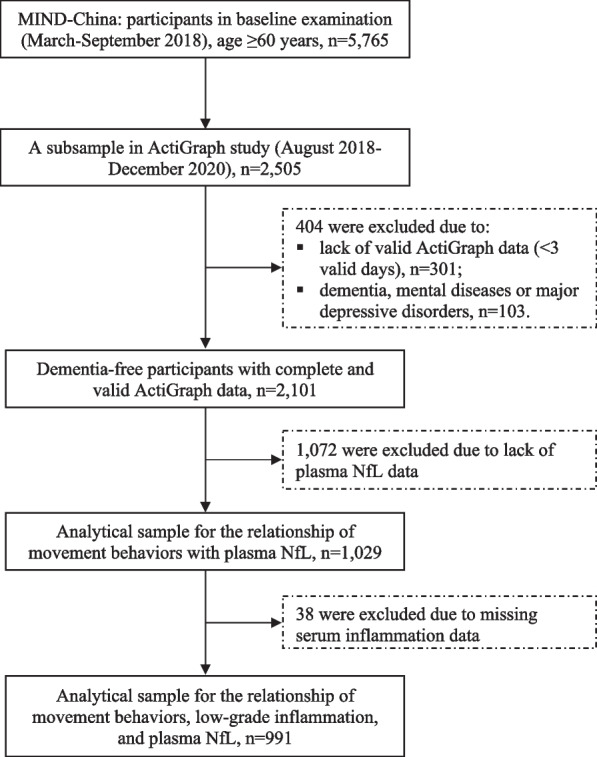
Flowchart of the study participants. Abbreviation: MIND-China, randomized controlled Multimodal INterventions to delay Dementia and disability in rural China; NfL, neurofilament light chain

This was a community-based cross-sectional study. The study sample was derived from participants in the baseline examination of the ongoing randomized controlled Multimodal INterventions to delay Dementia and disability in rural China (MIND-China), which is part of the World-Wide FINGERS Network [30, 31]. In brief, MIND-China is primarily aimed at testing whether the vascular intervention program (i.e., health education and monitoring and medical controls of high blood pressure, high blood glucose, and hyperlipidemia) and the multimodal intervention program (i.e., vascular intervention plus group exercise, personalized leisure activities, and computer-based cognitive training) are effective in maintaining cognitive and physical functioning in older adults. At baseline, MIND-China targets all registered residents who were aged ≥ 60 years by the end of 2017 and living in the 52 villages of Yanlou Town, Yanggu County, western Shandong Province. In March-September 2018, a total number of 5,765 residents (74.9% of all eligible persons) participated in the baseline examination. In August 2018-December 2020, a subsample of 2,505 participants in MIND-China underwent the ActiGraph examination. Of these, we excluded 404 participants due to insufficient wearing time (< 3 valid days) of ActiGraph (*n* = 301) and dementia, severe mental diseases or major depressive disorders (*n* = 103). Of the remaining 2,101 dementia-free participants, data on plasma NfL were available in a subsample of 1,029 persons. Among these, 38 were further excluded due to missing data on serum inflammatory biomarkers, leaving 991 persons for the analysis involving systemic low-grade inflammatory biomarkers. Figure 2 shows flowchart of the study participants.

### ActiGraph data collection and processing

Participants were instructed to wear an ActiGraph wGT3X-BT triaxial accelerometer (ActiGraph, LLC, Pensacola, FL) on their hip, affixed to an elastic belt, during all waking hours for seven consecutive days, and to remove it only for bathing [32, 33]. The accelerometer recorded data on counts per minute (CPM) and lasting time for each value of CPM. We analyzed data from the ActiGraph accelerometer for participants who wore the device for at least 10 h per day for a minimum of 3 days [34]. We defined SB, LPA, and MVPA as < 100, 100–1,040, and ≥ 1,041 CPM, respectively, according to previous reports [35]. Wearing seasons of accelerometer were categorized as spring, summer, autumn, and winter.

### Plasma NfL and serum cytokines

EDTA plasma was collected, aliquoted, and stored at -80ºC according to standard procedures until further analysis. In the biomarker subsample, plasma NfL concentration was measured on the SIMOA platform (Quanterix Corp, MA, USA) with the NF-light® advantage Kit following the manufacturer’s instructions (Wayen Biotechnologies Inc., Shanghai, China) [36]. Serum cytokines (e.g., IL-6, IL-8, TNF-α, and ICAM-1) were measured with commercially available meso scale discovery (MSD) V-PLEX® Proinflammatory Panel, as previously described [37].

### Measurement of covariables

Data on demographics, lifestyles, medical history, and use of medications were collected through face-to-face interview, clinical examinations, and laboratory tests [38]. All medications were classified and coded according to the Anatomical Therapeutic Chemical (ATC) Classification System [39]. Body mass index (BMI) was calculated as weight (kg) divided by height squared (m^2^) and obesity was defined as BMI ≥ 28 kg/m^2^ [40]. Education was categorized into illiterate (no formal schooling education), primary school, and middle school or above. Smoking and alcohol drinking status were categorized as never, former, and current smoking or drinking alcohol. Hypertension was defined as systolic pressure ≥ 140 mmHg or diastolic pressure ≥ 90 mmHg or current use of antihypertensive medications (ATC codes C02, C03, and C07-C09), diabetes as self-reported history of diabetes diagnosed by a physician or fasting blood glucose ≥ 7.0 mmol/L or current use of blood glucose-lowering medications (ATC code A10), and dyslipidemia as total cholesterol ≥ 6.22 mmol/L or low-density lipoprotein cholesterol ≥ 4.14 mmol/L or triglyceride ≥ 2.27 mmol/L or high-density lipoprotein cholesterol < 1.04 mmol/L or having received medications for high cholesterol or dyslipidemia (ATC code C10). Apolipoprotein E (*APOE*) genotyping was performed using multiple-polymerase chain reaction amplification and *APOE* genotype was dichotomized into carriers vs. non-carriers of the *APOE* ε4 allele [41].

### Statistical analysis

Characteristics of the study participants were presented with frequencies (%) for categorical variables and mean (standard deviation, SD) or median (interquartile range, IQR) for continuous variables depending on their distributions. We compared characteristics of the study participants by sex using the chi-square test for categorical variables, *t*-test for continuous variables with normal distribution, and Kruskal–Wallis test for those with skewed distribution.

Plasma NfL concentration was natural log-transformed before model fitting owing to skewed distribution. The association patterns of SB, LPA, and MVPA with plasma NfL were examined using RCS analysis. We ran the RCS models with 3 knots placed at the 10^th^, 50^th^, and 90^th^ percentiles to test if daily SB, LPA, and MVPA were linearly associated with plasma NfL [33]. If RCS analysis suggested a linear relationship, daily SB, LPA, and MVPA were analyzed as continuous variables using the general linear regression models. When the non-linear association was detected, we plotted the dose–response trajectories in RCS analysis for each outcome. Inflection points were defined as the number (approximately equal to an integer) of daily hours of SB, LPA, and MVPA at which the relationships with plasma NfL changes [42]. Then, we further analyzed the association of SB, LPA, and MVPA with plasma NfL after grouping the participants according to the respective inflection points. We reported the main results from three models: Model 1 was adjusted for age, sex, education, and ActiGraph wear season and daily wear time; Model 2 was additionally adjusted for *APOE* ε4 allele, smoking, alcohol intake, obesity, hypertension, dyslipidemia, diabetes, stroke, and coronary heart disease; and to assess the mutual impact of movement behaviors on the results, in Model 3, the analyses of MVPA and LPA were mutually adjusted for each other and the analyses of SB were adjusted for MVPA.

A composite score of the low-grade inflammatory biomarkers was calculated according to predefined clusters of conceptually related biomarkers, including IL-6, IL-8, TNF-a, and ICAM-1 [43]. Specifically, measures of serum cytokines were screened for outliers (above mean plus 3 SDs), log-transformed due to skewed distributions of original data (except TNF-α and ICAM), and then converted to a standardized z-score. The z-scores of individual cytokines (i.e., IL-6, IL-8, TNF-a, and ICAM-1) were averaged to yield a composite score for low-grade inflammation.

We used Stata Statistical Software, Release 15.0 (Stata Corp LLC., College Station, TX, USA) and R-3.6.3 for Windows (R Foundation for Statistical Computing, Vienna, Austria. https://www.R-project.org/) for all the analyses. Two-tailed *P* < 0.05 was considered to be statistically significant.

## Data Availability

The datasets used and/or analyzed during the current study are available from the corresponding author upon reasonable request and approval from the MIND-China Steering Committee at the Department of Neurology, Shandong Provincial Hospital, Jinan, Shandong, China.

## Abbreviations

Aβ: Amyloid beta
AD: Alzheimer’s disease
AIBL: The Australian Imaging, Biomarkers and Lifestyle study
*APOE*: Apolipoprotein E gene
BMI: Body mass index
CI: Confidential interval
CPM: Counts per minute
CSF: Cerebrospinal fluid
SB: Sedentary behavior
IFN-γ: Interferon gamma
ICAM-1: Intercellular cell adhesion molecule-1
IL-6: Interleukin-6
IL-8: Interleukin-8
IQR: Interquartile range
LPA: Light-intensity physical activity
MIND-China: Randomized controlled Multimodal INterventions to delay Dementia and disability in rural China
MCP-1: Monocyte chemotactic protein-1
MSD: Meso scale discovery
MVPA: Moderate-to-vigorous-intensity physical activity
NfL: Neurofilament light chain
PA: Physical activity
PET: Positron Emission Computed Tomography
RCS: Restricted cubic spline
SD: Standard deviation
SIMOA: Single molecule array
TNF-α: Tumor necrosis factor alpha
